# Severe Hepatotoxicity in Mushroom Poisoning by *Lepiota brunneoincarnata* from Complete Recovery to Liver Transplantation: A Case Series with Review on Liver Function Tests and Liver Histopathology

**DOI:** 10.1155/2024/2797712

**Published:** 2024-01-22

**Authors:** Mohammad Hossein Anbardar, Neda Soleimani, Kourosh Kazemi, Zahra Jafarpour, Mahsa Hasani, Sahand Mohammadzadeh, Parnia Torfehnezhad, Sedighe Jafarian, Mahsa Farhadi, Mina Salari Sardari

**Affiliations:** ^1^Department of Pathology, Shiraz Medical School, Shiraz University of Medical Sciences, Shiraz, Iran; ^2^Department of Pathology, Shiraz Transplant Center, Abu Ali Sina Hospital, Shiraz University of Medical Sciences, Shiraz, Iran; ^3^Department of Surgery, Shiraz Transplant Center, Abu Ali Sina Hospital, Shiraz University of Medical Sciences, Shiraz, Iran; ^4^Shiraz Transplant Center, Abu Ali Sina Hospital, Shiraz University of Medical Sciences, Shiraz, Iran

## Abstract

**Background:**

In spite of the scientific evidence supporting health advantages of mushrooms, some of them are seriously poisonous. The clinical picture of mushroom intoxication ranges from minor gastrointestinal symptoms to organ failure, such as liver failure and death.

**Method:**

We provided demographics, clinicopathological characteristics, applied treatments, and outcomes of mushroom poisoning by Lepiota species in a series of 18 cases that were referred from Kermanshah and Lorestan provinces to Abu-Ali-Sina Hospital, Shiraz, Iran. Clinical and paraclinical data were collected by taking history and reviewing of medical documents. Pathologic findings were extracted through a review of hematoxylin and eosin pathologic slides.

**Results:**

The patients were between the ages of 18 and 67 years, composed of ten females and eight males. The most frequent clinical manifestations were nausea and vomiting followed by abdominal pain. Four cases presented decreased consciousness on admission. One of them passed away. Three other cases underwent liver transplantation, two of them died after transplantation, and one fully recovered without any major issues. All instances had elevated ALT levels, which ranged from 44 to 9,140 IU/L (mean: 3259 ± 2476), with most of them also having concurrent AST elevations (mean: 1,361 ± 1,532). Only few patients had modest elevations in alkaline phosphatase. Total and direct bilirubin elevations up to 47.6 and 24 mg/dL, respectively, were found in most cases. Decreased total protein and albumin concentrations and increased BUN and creatinine levels were observed in some patients. In addition, some instances revealed increased LDH, increased WBC, decreased hemoglobin, and decreased platelet count. Most patients had increased prothrombin time; hematuria and positive stool occult blood were observed in few patients. Histopathologic examination of three explanted livers revealed massive necrosis with moderate to severe macrovesicular steatosis, significant ductular reaction, and parenchymal inflammation. Other patients followed a recovery process with a considerable drop in liver enzymes, especially ALT, during hospitalization utilizing conservative treatment. They had no liver problems or relevant issues after a two-year follow-up.

**Conclusion:**

In our study, highly elevated liver enzymes with a significantly high ALT/AST ratio were observed in cases of mushroom poisoning by Lepiota species, leading to fulminant liver failure and death in some cases. These laboratory findings were correlated with liver necrosis and macrovesicular steatosis in explanted livers.

## 1. Introduction

Mushrooms have recently gained popularity in the human diet due to their exquisite taste and texture, protein content, and an expanding body of scientific evidence supporting their health advantages. However, numerous mushrooms may prove toxic if eaten, and distinguishing between edible and poisonous species is often difficult. Mushroom poisoning has been reported from all around the world. Cyclopeptide-containing mushrooms are the most toxic species worldwide, responsible for 90-95% of human fatalities. With a high mortality rate, it is considered a medical emergency [1–4].

The clinical picture of patients with wild mushroom intoxication depends mainly on the type of ingested mushroom, which can range from minor gastrointestinal symptoms, such as abdominal pain, nausea, vomiting, and diarrhea, to organ failure and death. Hepatotoxicity and liver failure are associated with a small group of mushrooms [5].

In April and May 2018, about 1,200 persons from the western provinces of Iran were referred to hospital emergency rooms with symptoms of mushroom poisoning following ingestion of wild toxic mushrooms. These accidental poisonings happened to people who consumed wild mushrooms that they had personally collected from suburban areas, farms, gardens, hillsides, and forests or bought from local markets. *Lepiota brunneoincarnata* was the species responsible for this outbreak, confirmed by the Iranian Plant Pathology Research Institute, Tehran, Iran. The clinical presentations ranged from mild gastrointestinal problems (nausea, vomiting, diarrhea, and abdominal pain) to multiorgan failure, especially liver failure and death [6, 7].

This study is aimed at providing demographics, clinicopathological characteristics, and outcomes of mushroom poisoning in a series of 18 cases referred from Kermanshah and Lorestan provinces to Abu-Ali-Sina Hospital, Shiraz, Iran. We also review liver function tests and histopathological findings in mushroom poisoning.

## 2. Material and Methods

We retrospectively evaluated 18 mushroom poisoning patients admitted to Abu-Ali-Sina Hospital of Shiraz, Iran, between April and May 2018. The study was designed in accordance with the Declaration of Helsinki and carried out after obtaining approval from the Ethics Committee of Shiraz University of Medical Sciences. We requested and obtained informed consents from the patients for publishing the case details and the publication of the accompanying images.

The patients were suspected of mushroom poisoning based on their history of wild mushroom consumption, clinical symptoms, and paraclinical information. Age, gender, prior medical history, latent period, symptoms, duration of hospitalization, laboratory results (aspartate aminotransferase (AST), alanine aminotransferase (ALT), alkaline phosphatase (ALP), total and direct bilirubin, total protein, albumin, blood urea nitrogen (BUN), creatinine, lactate dehydrogenase (LDH), white blood cell (WBC), hemoglobin, platelet count, prothrombin time (PT), urinalysis, and stool occult blood (OB)), applied treatments, and outcomes were assessed. Demographic, clinical, and laboratory data were collected by taking history and reviewing medical documents. Pathologic findings were extracted by reviewing hematoxylin and eosin (H&E) pathologic slides.

### 2.1. Statistical Analyses

SPSS (Statistical Package for Social Sciences) for Windows 10.0 was used for the statistical analysis. Quantitative data were compared using the Mann–Whitney *U* test as well as definitive tests like standard deviation analysis. Chi-square and Fisher's exact Chi-square test were used in comparing qualitative data.

## 3. Results

### 3.1. Clinical Features and Laboratory Data

Table 1 summarizes the clinical characteristics of all patients. The patients were between the ages of 18 and 67 years (median: 45.5). Ten (55.5%) cases were female, and eight (44.5%) were male. A varied quantity of wild mushrooms were devoured by them. There was a 6- to 12-hour latent phase. For those who were alive, the average hospital stay was five days.

Fourteen (78%) patients had no relevant prior medical history. However, one of them was in the second trimester of pregnancy. The other four cases had histories of ischemic heart disease (IHD), hypertension (HTN), diabetes mellitus (DM), and opium addiction. Two cases had histories of alcohol use. The most frequent clinical manifestations were nausea and vomiting (88.8%) and abdominal pain (50%). Four cases (numbers 2, 3, 5, and 6) presented decreased consciousness on admission. One of them (number 3) passed away on the second day. Three other cases underwent liver transplantation. Cases 2 and 5 died two and six days after transplantation, respectively, whereas case number 6 fully recovered without any major issues.

Serologic tests for hepatitis B and C viruses were negative in all cases. Tables 2 and 3 provide a summary of initial laboratory data. All instances had elevated ALT levels, which ranged from 44 to 9,140 IU/L (mean: 3259 ± 2476), with most of them also having concurrent AST elevations (mean: 1,361 ± 1,532). Only four patients had modest elevations in alkaline phosphatase (mean: 257 ± 87). Total and direct bilirubin elevations up to 47.6 (mean: 9.1 ± 11.6) and 24 mg/dl (mean: 4.3 ± 5.7), respectively, were found in all cases, except for one. Eleven cases had decreased total protein concentration (mean: 5.6 ± 0.7), and eight showed concomitant albumin decrease (mean: 3.5 ± 0.6). Four cases had increased BUN, up to 94 mg/dL, and three had concurrently increased creatinine, up to 4.9 mg/dL. Nine instances underwent LDH testing, and five showed increased results. Increased WBC, decreased hemoglobin, and decreased platelet counts were seen in 27%, 33%, and 39% of patients, respectively. 78% of patients had increased PT. Eight cases showed hematuria, and four were positive among nine cases tested for stool OB.

### 3.2. Histopathological Features

**Figure 1:**
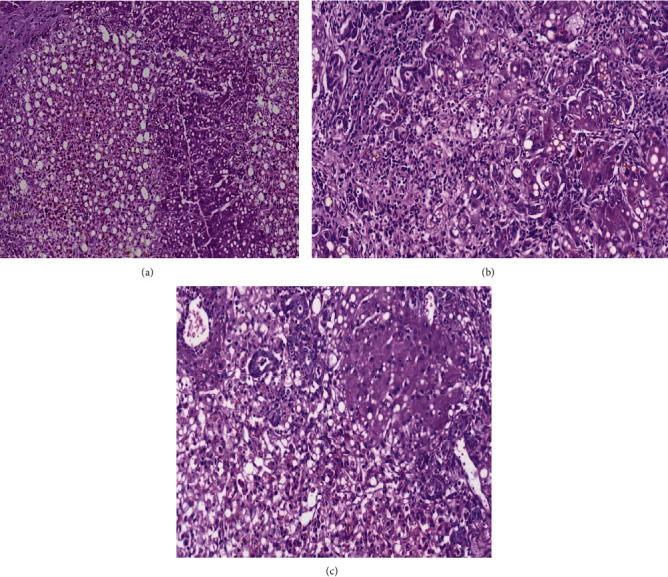
Histologic examination of explanted livers showing significant macrovesicular steatosis (a), submassive necrosis with ductular reaction (b), and inflammatory cell (macrophages and neutrophil) infiltration (c).

Three explanted livers (cases 2, 5, and 6) were evaluated in the pathology department. Macroscopically, all three cases were brown and smooth with a soft consistency and no discrete nodule. Histopathologic examination revealed massive necrosis with moderate to severe macrovesicular steatosis (Figure 1). There were also significant ductular reactions and parenchymal inflammation.  Table 4 provides macroscopic and microscopic descriptions of explanted livers. All attached gallbladders were unremarkable.

### 3.3. Follow-Up

**Figure 2:**
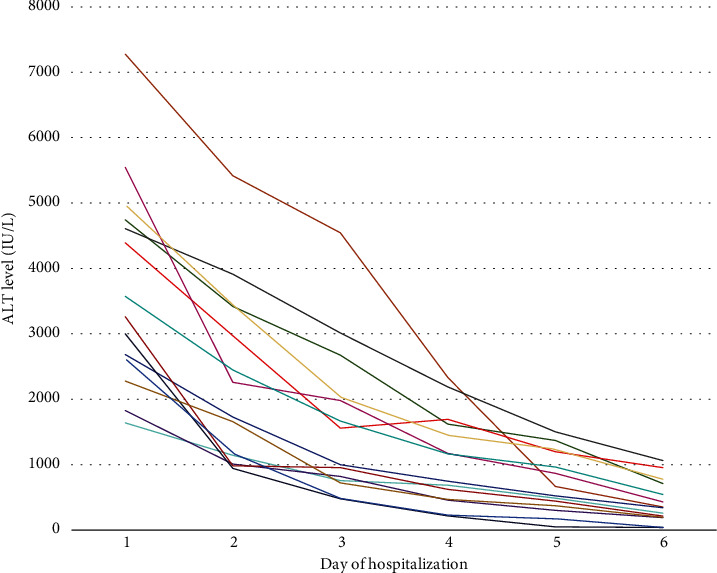
Serial ALT level of patients during hospitalization.

All patients followed a recovery process with a considerable drop in liver enzymes, especially ALT, during hospitalization utilizing conservative treatment, except for those who underwent liver transplantation and those who passed away (Figure 2). Furthermore, they had no liver problems or relevant issues after a two-year follow-up.

## 4. Discussion

Although there are approximately 140,000 mushroom species worldwide, just 2,000 are accepted as safe for human consumption. Approximately 100 mushroom species lead to toxicity in humans. Most of the time, nontoxic and poisonous mushrooms grow nearby; unfortunately, many mushrooms are difficult to identify even by a trained mycologist. Accurate estimates of worldwide poisoning by toxic mushrooms are difficult to establish due to the lack of case reporting in hospital emergency rooms [8, 9].

Toxic mushrooms can be grouped based on their toxic components: cyclopeptides, gyromitrin, muscarine coprine, isoxazoles, orellanine, psilocybin, and gastrointestinal irritants. From these, cyclopeptide-containing mushrooms are the most toxic species worldwide, responsible for 90-95% of human fatalities. The main toxic agents are amatoxins that are present in three genera: Amanita (mainly *A. phalloides*), Lepiota (*L. brunneoincarnata* as the most frequently reported), and Galerina (*G. marginata* as the most common) [10–12]. Amatoxin inhibits RNA polymerase II, resulting in deficient protein synthesis and cell death. Cells with high metabolism are mainly affected: hepatocytes, cells of the proximal tubules of the kidneys, and intestinal mucosa. Amatoxins are thermostable, so cooking or freezing does not alter their toxicity [12]. *A. phalloides* is responsible for most fatal cases due to mushroom poisoning. Some Lepiota species are also very toxic. They are less frequently involved in human poisoning than Amanita species. In addition to amatoxin, they contain amanitin [10–13].

Based on key clinical characteristics, a recent study proposed six main categories of mushroom poisoning, including cytotoxic mushroom poisoning (primary hepatotoxicity or nephrotoxicity), neurotoxic mushroom poisoning, myotoxic mushroom poisoning, metabolic and endocrine-related toxicity mushroom poisoning, gastrointestinal irritant mushroom poisoning, and other adverse reactions to mushrooms. However, numerous mushroom poisoning syndromes share some clinical characteristics, most notably gastrointestinal symptoms [14].

Toxins like amatoxins that produce potentially fatal hepatotoxicity and have a specific constellation of clinical features are included in the category of cytotoxic mushroom poisoning with primary hepatotoxicity. *A. phalloides* and Lepiota species are well-known examples. Clinically, this kind of poisoning manifests with a delayed onset of prehepatotoxic symptoms, starting with frequent severe gastrointestinal effects (nausea, vomiting, diarrhea, abdominal pain, and secondary dehydration) at around 9-12 hours after ingestion, though occasionally developing as early as six hours after ingestion, lasting up to 36 hours, followed by improvement. This is followed by about day 3 with obvious signs of hepatotoxicity, such as abnormal laboratory results (liver function and coagulation), progressing in severe cases to full liver failure (coagulopathy, bleeding, and hepatic encephalopathy), frequently associated with renal failure, and ending in death from hepatic failure at about day 7, unless preventive measures prove effective.

Based on the clinical signs and species of the mushroom, mushroom poisoning is categorized into low, medium, and high risk categories in order to meet the goals of a graded diagnosis, hierarchical treatment, and prompt patient referral [15, 16]. Patients with amatoxin-induced liver failure have received a variety of nonspecific treatments, such as gastric lavage and the administration of activated charcoal, which lowers the amount of toxins absorbed by inhibiting or decreasing enterohepatic circulation. The most commonly used particular treatment strategies for patients with amatoxin poisoning have been penicillin G, silybin, and N-acetylcysteine (NAC). However, none has been conclusively demonstrated to have great clinical efficacy; therefore, a substantial mortality rate (10-30%) is still confirmed [11, 17–21].

In extreme cases, the sole option for survival may be a liver transplant, which is occasionally either not possible or medically problematic. Dialysis may be necessary if renal insufficiency is severe [5, 14]. Patients with liver failure must be moved as soon as possible to a hospital that has a liver transplantation center. Liver transplantation should be used if conservative treatment is ineffective. Survival depends on the level of hepatic destruction, the capacity of surviving liver cells to recover, and the management of complications that may arise during intoxication. Liver transplantation has dramatically improved the survival rate and remains the cornerstone of treatment in some patients with fulminant hepatic failure. Organ transplantation requires available organ donation, so it is not always easy, and it is also a costly and risky procedure [22–24].

*L. brunneoincarnata* was the primary culprit in this mushroom outbreak [7]. Menier made the first observations about Lepiota toxicity as early as 1891 [25]. Most reported cases of fatal poisoning were related to the species *Lepiota helveola*. As mycological knowledge has advanced, more Lepiota species with comparable toxicity have been described more recently [26]. Even though radioimmunologic methods and thin-layer chromatography revealed a discrepancy regarding the level of amanitin in Lepiota and Amanita species, chemical analysis revealed the presence of alpha amanitin and occasionally gamma amanitin, but no beta amanitin or phallotoxins in Lepiota as opposed to Amanita [13, 27].

Lepiota species is less studied and reported on than Amanita species, specifically regarding its hepatotoxicity and histopathological abnormalities.

In nearly all of our cases, hepatotoxicity and hepatic failure were confirmed by a significant rise in ALT up to 9,140 IU/L (with a peak value on the fifth day after ingestion), increased ALT/AST ratio, increased bilirubin (both direct and indirect components), and increased INR. As in earlier research, there was no significant rise in alkaline phosphatase levels [28]. This pattern of liver injury is similar to Amanita species poisonings, whereas few previous reports of Lepiota species only mentioned increased ALT and ALT/AST ratios [13, 29, 30]. Furthermore, few prior publications of Amanita poisonings have mentioned fatty changes in the liver in severe instances, and most of these fatty changes were described by radiology without pathologic confirmation [7, 26, 29, 31]. In several of our patients, concurrent hematuria and elevated BUN/creatinine suggested nephrotoxicity, and in some cases, aberrant LDH, WBC count, hemoglobin, and platelet counts raised suspicions of a hemolytic process. A few cases also revealed stool OB, a finding which was also present in an earlier report of Lepiota species poisoning [32].

## 5. Conclusion

In mushroom poisoning, there are various clinical presentations and pathologic changes depending on the ingested species. Lepiota species are rare causes of fatal mushroom poisoning. Despite the fact that Amanita and Lepiota are two distinct species, the likelihood of liver failure seems to be essentially the same. In our study, highly elevated liver enzymes with a significantly high ALT/AST ratio were observed in cases of mushroom poisoning by Lepiota species, leading to fulminant liver failure and death in some cases. These laboratory findings were correlated with liver necrosis and steatosis in explanted livers.

## Figures and Tables

**Table 1:** Demographic and clinical data of cases with mushroom poisoning.

Case no.	Age (y/o)	Sex	Latent period (hrs)	Admission days	Past history	Clinical presentation	Medication	Outcome
1	30	M	8	10	Opium addict/alcohol drinker	N/V, abdominal pain	Ondansetron/pantoprazole Lactulose	Recovery
2	55	F	7	13	DM/HTN	N/V, diarrhea, and decreased LOC	Pantoprazole/lactulose Metronidazole/NAC Cefotaxime	Expired after liver transplantation
3	47	F	6	8	Nonsignificant	N/V, abdominal pain, and decreased LOC	Lactulose Rifaximin	Expired (after 2 days of admission)
4	32	F	10	6	Nonsignificant	N/V, abdominal pain	Ondansetron/pantoprazole Lactulose	Recovery
5	67	M	6	5	IHD/alcohol drinker	Decreased LOC	Pantoprazole/lactulose Cefotaxime/metronidazole Meropenem/vancomycin NAC	Expired after liver transplantation
6	49	M	7	49	Nonsignificant	Decreased LOC	Pantoprazole/imipenem	Recovery after liver transplantation
7	55	F	6	6	Nonsignificant	N/V, Lethargy/icterus	Ondansetron/pantoprazole Lactulose/metronidazole Ceftriaxone	Recovery
8	21	F	8	7	Pregnant GA: 20 (wks)	N/V	Pantoprazole/lactulose Ceftriaxone	Recovery
9	66	F	9	4	Nonsignificant	N/V, abdominal pain	Ondansetron/pantoprazole Lactulose/NAC	Recovery
10	40	F	8	7	Nonsignificant	N/V, diarrhea	Ondansetron/pantoprazole Lactulose/metronidazole	Recovery
11	52	M	11	5	Nonsignificant	N/V, abdominal pain	Ondansetron/lactulose	Recovery
12	30	F	10	5	Nonsignificant	N/V, abdominal pain	Ondansetron/lactulose	Recovery
13	37	M	10	2	Nonsignificant	N/V	Ondansetron/lactulose	Recovery
14	44	M	8	3	Nonsignificant	N/V, abdominal pain	Ondansetron/lactulose	Recovery
15	50	M	12	4	Nonsignificant	N/V	Ondansetron/lactulose	Recovery
16	59	M	12	6	DM	N/V	Ondansetron/lactulose	Recovery
17	28	F	10	5	Nonsignificant	N/V, abdominal pain	Ondansetron/lactulose	Recovery
18	18	F	10	3	Nonsignificant	N/V, abdominal pain	Ondansetron/lactulose	Recovery

N/V: nausea and vomiting; DM: diabetes mellitus; HTN: hypertension; LOC: level of consciousness; IHD: ischemic heart disease; GA: gestational age; NAC: N-acetyl cysteine.

**Table 2:** Liver function tests of patients.

Case no.	AST (0-40) (IU/L)	ALT (0-40) (IU/L)	ALP (80-306) (IU/L)	Total bilirubin (0.2-1.3) (mg/dL)	Direct bilirubin (0.1-0.3) (mg/dL)	Total protein (6-8.8) (gr/dL)	Albumin (3.5.5) (gr/dL)
1	5301	7277	237	5.4	2.3	5.5	3.7
2	4104	9140	406	4.1	2.5	4.6	3.2
3	162	3450	343	47.6	24	6.6	2.8
4	109	1638	92	2	0.6	6	3.7
5	1736	4500	284	17.8	6.8	6.1	3
6	1800	4300	289	12	7.9	4.1	2.3
7	1449	4743	362	5.7	3.3	4.8	3
8	675	1827	166	2.3	1.2	5	3.1
9	582	2682	280	2.04	0.9	6.3	4.2
10	3456	4608	216	21.4	8.3	4.7	3.2
11	522	4392	366	10.9	6	5.7	3.3
12	1880	3260	144	1.1	0.2	5.4	3.2
13	27	3000	250	1.8	0.4	6.2	4.4
14	237	456	134	1.5	0.4	6.5	4.4
15	2817	5544	315	2.1	0.8	5.8	3.9
16	252	2277	305	23	9.9	6.1	3.9
17	432	3573	154	1.5	0.4	5.2	3.8
18	900	4941	277	2.9	1.1	5.7	3.7

AST: aspartate aminotransferase; ALT: alanine aminotransferase; ALP: alkaline phosphatase.

**Table 3:** Other laboratory data of cases.

Test (reference range) unit	BUN (6–20) (mg/dL)	Creatinine (0.5-1.3) (mg/dL)	LDH (200-400) (mg/dL)	WBC 4.5–11 (×10^3^/*μ*L)	Hemoglobin Men: 14–18 Women: 12-16 (g/dL)	PLT 150–450 (×10^3^/*μ*L)	Prothrombin time (10–14) (second)	Urinalysis	Stool OB
Case 1	5	0.9	4310	5.8	14.5	152	17	NL	-ve
Case 2	29	2	285	14.3	8.9	262	15	NL	-ve
Case 3	26	1.2	NA	22.8	6.7	49	18	NL	-ve
Case 4	11	0.9	NA	10.6	11.9	353	15	NL	NA
Case 5	53	4.9	NA	13.2	13.3	51	25	NL	-ve
Case 6	94	2.7	NA	6.2	10.7	27	13	NL	NA
Case 7	18	0.8	NA	8	14.1	176	13	Blood: 3+	NA
Case 8	4	0.6	NA	13	12.5	244	13	Blood: 3+	2+
Case 9	9	1	2196	8.5	15	245	21	NL	NA
Case 10	11	0.6	NA	10.1	14.5	50	18	Blood: 3+	NA
Case 11	17	1.3	NA	14.3	16.7	85	18	NL	Trace
Case 12	11	0.9	548	8.9	12.9	240	15	Blood: 3+	NA
Case 13	13	0.9	265	5.4	16.2	212	14	NL	NA
Case 14	11	0.8	276	11	16.7	335	13	Blood: 3+	1+
Case 15	20	0.9	562	10.1	15	167	17	NL	-ve
Case 16	22	0.6	3000	13	11.4	121	15	Blood: 1+ Bilirubin: 1+ Urobilinogen: 2+	NA
Case 17	6	0.8	317	7.4	15.4	119	15	Blood: 2+	NA
Case 18	3	0.6	NA	4.7	15.3	164	16	Blood: 3+	Trace

BUN: blood urea nitrogen; LDH: lactate dehydrogenase; WBC: white blood cell; PLT: platelet; OB: occult blood; NL: normal; NA: not available.

**Table 4:** Macroscopic and microscopic features of explanted livers.

Case no.	Macroscopic features			Microscopic features			
	Liver size (cm)	Capsular surface	Cut surface	Necrosis (%)	Hemorrhage (%)	Macrovesicular steatosis (%)	Microvesicular steatosis (%)
Case 2	26 × 16 × 11	Smooth Brown-gray	Smooth Brown No nodule	10%	10%	70%	5%

Case 5	20 × 16 × 10	Smooth Brown	Smooth Brown No nodule	30%	5%	50%	10%

Case 6	21 × 15 × 9	Smooth Brown	Smooth Dark Brown No nodule	30%	5%	40%	10%

## Data Availability

All data generated or analysed during this study are included in this published article.

## Abbreviations

AST: Aspartate aminotransferase
ALT: Alanine aminotransferase
ALP: Alkaline phosphatase
BUN: Blood urea nitrogen creatinine
LDH: Lactate dehydrogenase
WBC: White blood cell
PT: Prothrombin time
OB: Stool occult blood
H&E: Hematoxylin and eosin.
